# Big data and artificial intelligence in animal nutrition: a new era of precision feeding

**DOI:** 10.1007/s11250-026-05077-8

**Published:** 2026-05-12

**Authors:** Purbita Devi, Sabuj Kanti Nath, Brishti Barua, Tithe Saha

**Affiliations:** 1 Faculty of Veterinary Medicine, Chattogram Veterinary and Animal Sciences University, Khulshi, 4225 Bangladesh; 2 College of Science and Engineering, James Cook University, Townsville, QLD 4811 Australia; 3 Department of Animal Nutrition, Faculty of Veterinary, Animal and Biomedical Sciences, Khulna Agricultural University, Khulna, 9100 Bangladesh; 4 Department of Medicine and Surgery, Faculty of Veterinary Medicine, Chattogram Veterinary and Animal Sciences University, Khulshi, 4225 Bangladesh; 5 Department of Physiology, Faculty of Veterinary, Animal and Biomedical Sciences, Khulna Agricultural University, Khulna, 9100 Bangladesh

**Keywords:** Climate resilience, Machine learning, Microplastics, Multi-omics, Precision nutrition

## Abstract

The convergence of Big Data and Artificial Intelligence (AI) is redefining animal nutrition by enabling precision feeding systems that are individualized, data-driven, and sustainability-oriented. This review synthesizes recent advances in multi-omics technologies, sensor-based monitoring, and machine learning applications across feed formulation, health surveillance, and production optimization. Precision feeding in pigs has been shown to reduce production costs by more than 8%, decrease protein and phosphorus intake by approximately 25%, lower nutrient excretion by up to 40%, and reduce greenhouse gas (GHGs) emissions by 6%, while maintaining or improving performance. In dairy systems, precision feed management strategies have achieved approximately 9.7% lower dietary crude protein levels, 14% reductions in manure nitrogen excretion, and annual net income gains of USD 137 per cow. AI-driven models have enhanced prediction of milk yield, feed conversion ratio (R² = 0.74), and residual feed intake (R² = 0.76), while enabling 96.26% accuracy in detecting microplastics in poultry feed. Integration of genomic, phenotypic, and sensor-derived datasets supports real-time monitoring, with wearable and IoT technologies transforming livestock management through continuous tracking of feeding behavior, emissions, and welfare indicators. Despite significant progress, current systems remain constrained by data heterogeneity, limited interoperability, and insufficient prescriptive decision-support frameworks. This article identifies methodological, technological, and adoption-related gaps, while highlighting future directions including nutrigenomics- and metagenomics-informed diet design, adaptive precision nutrition, and cost-effective solutions for smallholder systems. Collectively, these innovations establish Big Data and AI-enabled precision nutrition as a cornerstone of sustainable livestock production, advancing food security, climate resilience, and ethical animal management.

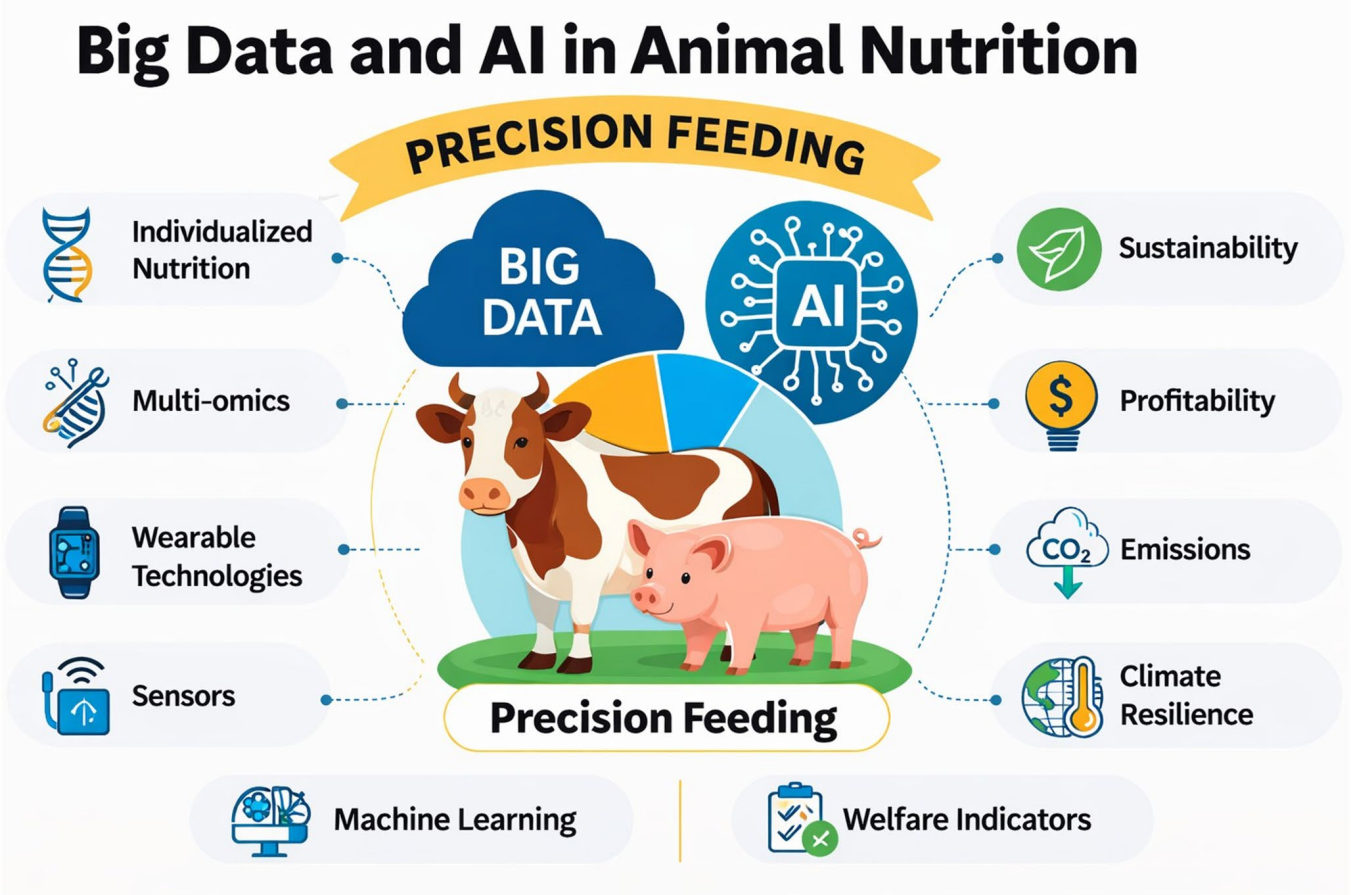

## Introduction

The integration of Big Data and Artificial Intelligence (AI) is transforming animal nutrition by advancing data-driven decision making and enabling individualized feeding strategies. Within the broader framework of precision livestock farming (PLF), these technologies enhance the capacity to monitor, analyze, and optimize animal performance in real time. As global livestock systems face increasing pressure from climate change, resource constraints, and rising demand for animal-source foods, precision feeding has emerged as a central strategy for improving nutrient-use efficiency while minimizing environmental impact. Advances such as precision fermentation enable scalable production of high-quality proteins and nutrients using engineered microorganisms (Ajayeoba and Ijabadeniyi 2025), while IoT-enabled sensors, drones, hyperspectral imaging, and AI-driven analytics enhance real-time monitoring of agricultural systems. However, persistent challenges in calibration, interoperability, data privacy, and adoption highlight the need for innovations in miniaturization, wireless communication, and energy efficiency (Aarif et al. 2025).

Nutrition remains a pivotal driver of sustainable and ethically aligned livestock production. Novel feed constituents, precision nutrition, and sustainability-oriented strategies are gaining prominence in response to regulatory, economic, and consumer pressures (Sonia et al. 2023). The integration of Big Data and machine learning (ML) into nutritional science has been particularly significant for feed authentication, quality assurance, and safety. Portable detection devices, miniaturized spectrometers, and biosensors linked to AI models enable real-time monitoring of contaminants with enhanced selectivity, sensitivity, and stability (Hassan et al. 2025; Liang et al. 2025; Pervaiz et al. 2025). Similarly, optical non-destructive techniques combined with ML provide scalable food quality assessments, overcoming the limitations of conventional destructive testing (X. Wang et al. 2025a, b). AI methods, including supervised, unsupervised, and reinforcement learning, are increasingly applied to spectral, imaging, and behavioral datasets for aflatoxin detection and food safety monitoring (Deshmukh et al. 2025). ML has also improved dietary assessment tools and origin-tracing methods, though challenges remain in interpretability, standardization, and integration across platforms (Li et al. 2025).

In animal nutrition, Big Data and AI are applied from feed formulation to health monitoring and production optimization, enabling the development of precision feeding systems tailored to individual animal requirements. A scoping review of 151 studies revealed that dairy decision-support research is recent (mean age 5.95 years), primarily targeting milk prediction (29%), lameness (26%), and mastitis detection (13%), using historical datasets (70%) with artificial (47%) and convolutional neural networks (24%), while prescriptive (3%), real-time (25%), simulation (4%), and stochastic models (5%) remain underutilized (Palma et al. 2025). Machine learning has improved milk yield and sustainability (Akintan et al. 2025), detected microplastics in poultry feed with 96.26% accuracy (Liu et al. 2025), and predicted mink growth and efficiency traits, ADG (R² = 0.71), FCR (R² = 0.74), RFI (R² = 0.76), with sex as the strongest predictor (Shirzadifar et al. 2025). These examples highlight how AI-driven analytics advance precision feeding through optimized nutrient delivery and performance prediction.

Despite these advances, critical gaps remain. Most studies emphasize predictive modeling based on historical data, while prescriptive and real-time decision-support systems are underdeveloped. Data heterogeneity, lack of standardization, and limited interoperability constrain the integration of nutritional, environmental, and behavioral datasets. Adoption at the farm level is further restricted by high costs, privacy concerns, and limited model interpretability, while many PLF technologies remain at the testing stage with insufficient scalability across production systems (Brassó et al. 2025).

Taken together, these developments mark a paradigm shift toward precision feeding systems powered by Big Data and AI. This review critically evaluates applications of AI and Big Data in feed formulation, precision feeding, and food quality and safety, identifies technological, methodological, and adoption-related gaps, examines sustainability-oriented strategies within regulatory and consumer contexts, and outlines future pathways for developing scalable, interpretable, and ethically aligned nutrition systems to advance sustainable livestock production.

Accordingly, although this review addresses a range of emerging technologies and interdisciplinary themes, its primary focus is on precision feeding and nutrient management in animal nutrition. Big Data analytics, AI, ML, sensor technologies, and omics approaches are treated as enabling tools that support nutrition-driven decision-making rather than independent objectives. This perspective aligns with established definitions of precision feeding, which emphasize the dynamic matching of nutrient supply to changing animal requirements to improve feed efficiency and reduce nutrient losses (González et al. 2018; Pomar and Remus 2019; Zuidhof 2020). Related topics, including feed safety, antimicrobial resistance, and sustainability, are therefore discussed only insofar as they directly affect nutrient utilization, feeding efficiency, or diet formulation, ensuring depth in core nutritional concepts while highlighting the applied relevance of data-driven precision feeding systems.

## Literature search and selection framework

### Literature search strategy

A comprehensive and systematic literature search was conducted to identify peer-reviewed studies examining the applications of Big Data, AI, machine learning, and digital technologies in animal nutrition and precision feeding. The search was performed across four major scientific databases: Web of Science, Scopus, PubMed, and Google Scholar. To refine the search, a combination of Boolean operators and keywords was used. The main keywords used were: (“big data” OR “artificial intelligence” OR “machine learning” OR “deep learning” OR “data analytics”) AND (“animal nutrition” OR “precision nutrition” OR “precision feeding” OR “feed formulation”) AND (“livestock” OR “ruminant” OR “cattle” OR “dairy cow” OR “beef cattle” OR “pig” OR “poultry”) AND (“omics” OR “genomics” OR “metagenomics” OR “nutrigenomics” OR “phenomics” OR “sensor” OR “IoT”).

### Inclusion and exclusion criteria

To ensure the relevance and quality of the selected literature, specific inclusion and exclusion criteria were applied. Eligible studies were limited to peer-reviewed journal articles published in English between 2019 and 2025, including original research, systematic reviews, and high-quality narrative reviews. In addition to studies directly focused on livestock species (e.g., ruminants, pigs, and poultry), the review also included research covering a broader spectrum of applications of Big Data, artificial intelligence, machine learning, sensor-based technologies, and multi-omics approaches across animal science, agriculture, food systems, and related biological domains, provided that the findings were conceptually or methodologically relevant to nutrition, feeding strategies, health monitoring, sustainability, or decision-support frameworks. Studies were excluded if they lacked a clearly defined methodological or analytical framework, were published in non-peer-reviewed formats (e.g., conference abstracts, editorials, commentaries, or theses), did not provide full-text access, or addressed digital or computational technologies without a substantive and transferable link to nutrition, feeding management, or agri-food systems.

### Article selection and data extraction

**Fig. 1:**
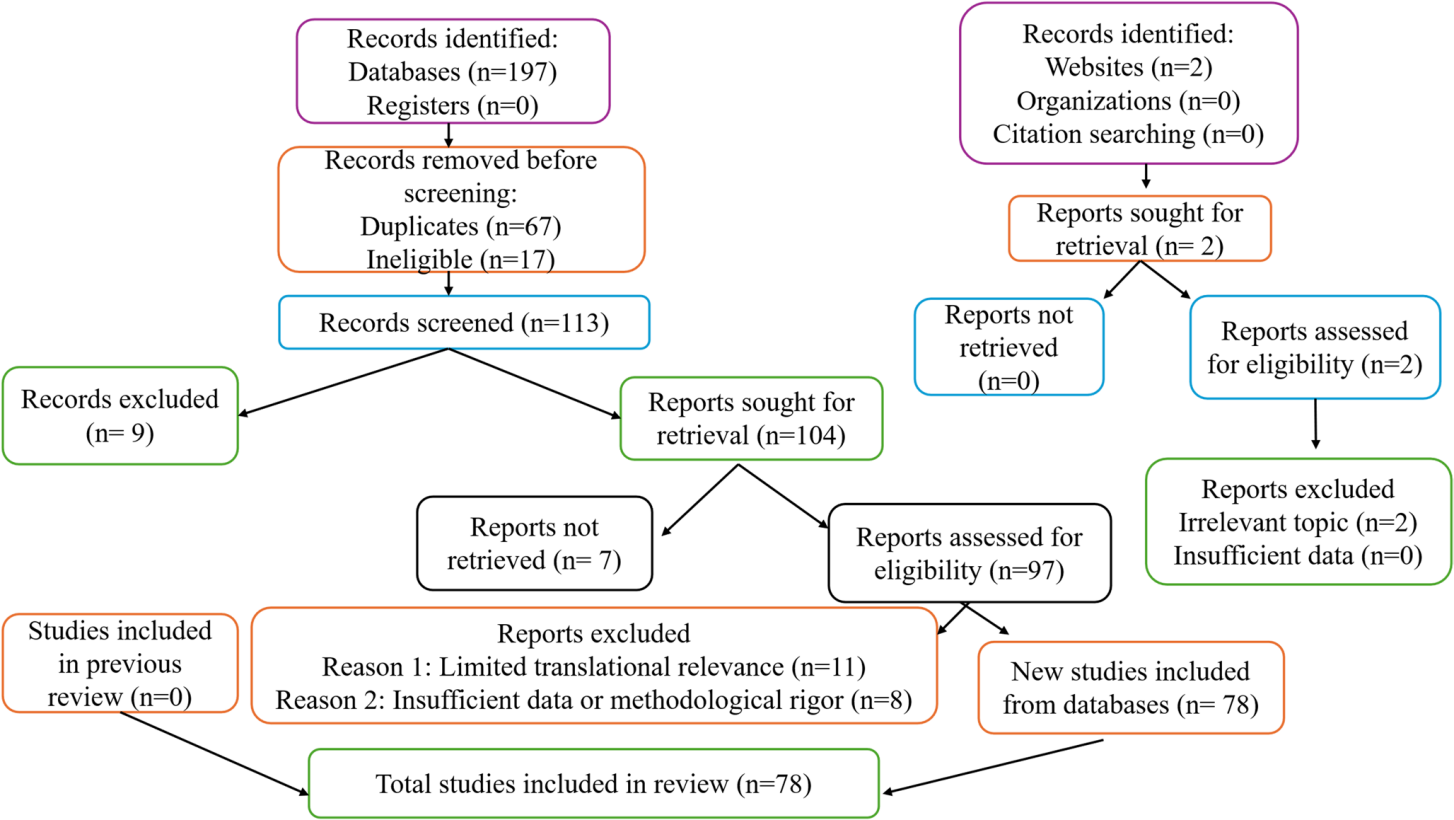
PRISMA flow diagram illustrating the literature search, screening, eligibility assessment, and study inclusion process for the systematic review. Records were identified from electronic databases, registers, websites, and organizational sources. After removal of duplicates and ineligible records, studies were screened, assessed for eligibility, and excluded based on predefined criteria. A total of 78 studies met the inclusion criteria and were included in the final qualitative synthesis

After collecting the preliminary findings, duplicate studies were removed using EndNote reference management software (ClarivateTM, Philadelphia, PA, USA). Subsequently, to identify relevant studies, titles and abstracts were screened, and full-text papers were assessed based on the inclusion criteria mentioned above. Initially, a total of 197 studies were screened, out of which 78 met the predefined inclusion and exclusion criteria and were included in this review. These selected studies were then narratively synthesized, focusing on their main findings, research approaches, and relevance to the topic. In this review, to illustrate the selection process, we adhered to the Preferred Reporting Items for Systematic Reviews and Meta-Analyses (PRISMA) framework (Fig. 1).

### Comparative synthesis and interpretation of evidence

This review synthesizes evidence from peer-reviewed studies using a narrative and comparative analytical approach rather than primary data analysis. Reported quantitative outcomes (e.g., percentage reductions, R² values, and accuracy metrics) were extracted directly from original studies and interpreted descriptively to evaluate the performance of Big Data and AI-driven precision nutrition systems. Emphasis was placed on consistency of effect sizes, methodological rigor, and relevance across livestock species and production systems. Statistical indicators reported in the source studies were used to support comparative insights without reanalysis, ensuring accuracy and transparency in interpretation.

## Fundamentals of precision animal nutrition

Precision animal nutrition is founded on the principle of supplying nutrients precisely according to the individual requirements of each animal, using real-time monitoring, sensor-based technologies, and predictive modeling to optimize performance and efficiency. In pigs, precision feeding systems such as InraPorc leverage temporal and individual variability in nutrient needs, achieving over 8% lower production costs, 25% reductions in protein and phosphorus intake, 40% less nutrient excretion, and a 6% decrease in GHGs emissions, while enhancing animal welfare (Gaillard et al. 2020; Pomar and Remus 2019, 2023). Electronic sow feeders exemplify individualized delivery by supporting higher piglet weaning weights and reduced sow bodyweight loss and feed cost per kilogram of piglet weaned without altering overall feed intake, milk composition, or metabolic status (Aparicio et al. 2024). Similarly, in poultry, precision nutrition improves feed efficiency (*p* < 0.001), increases apparent metabolizable energy between days 25–27 (*p* = 0.002), and reduces weight variation (*p* < 0.026), while maintaining growth performance and feed cost neutrality (Nawab et al. 2025). These applications demonstrate how real-time data integration enables nutrient supply to match dynamic physiological requirements.

Within this framework, precision feeding represents the practical application of precision animal nutrition within PLF, in which nutrient supply is automatically and continuously adjusted in real time at the individual or small-group level using sensor-derived data and predictive models to optimize nutrient utilization while maintaining animal performance (Pomar et al. 2019). This individualized approach improves nutrient use efficiency and substantially reduces excess nutrient excretion and associated environmental losses (González et al. 2018; Pomar and Remus 2023; Zuidhof 2020). Recent advances in sensor technologies, automated feeding systems, and decision-support tools have facilitated the translation of nutritional theory into real-time feeding decisions, positioning precision feeding as a foundational component of data-driven livestock production systems.

In dairy cattle, precision feeding strategies that tailor dietary protein to individual requirements, reducing urinary nitrogen and milk urea nitrogen without affecting milk yield, though localized monitoring has reported increases in methane (CH₄: +55%) and carbon dioxide (CO₂: +15%) emissions (Morey et al. 2023). In the United States, long-term implementation of Precision feed management has lowered dietary crude protein by ~ 9.7%, reduced manure nitrogen excretion by ~ 14%, and increased net income per cow by USD137 annually; over two decades, these strategies have achieved a ~ 10.8% reduction in dietary nitrogen, ~ 40% higher milk yield per cow, ~ 8.1% decrease in manure nitrogen, and a ~ 19% decline in carbon footprint (Chase and Fortina 2023). By integrating real-time sensor data with nutritional simulation models and advanced analytics, precise feeding systems enable accurate prediction of individual nutrient intake, metabolism, environmentally relevant emissions (including enteric CH₄ and CO₂, as well as nitrogen losses through urine and manure), and production outcomes. This data-driven integration exemplifies the core principles of precision nutrition: individualized nutrient supply, optimization of feed efficiency, reduction of nutrient excretion and GHGs emissions, enhancement of environmental sustainability, economic viability, and welfare-oriented management (González et al. 2018; Zuidhof 2020).

**Fig. 2:**
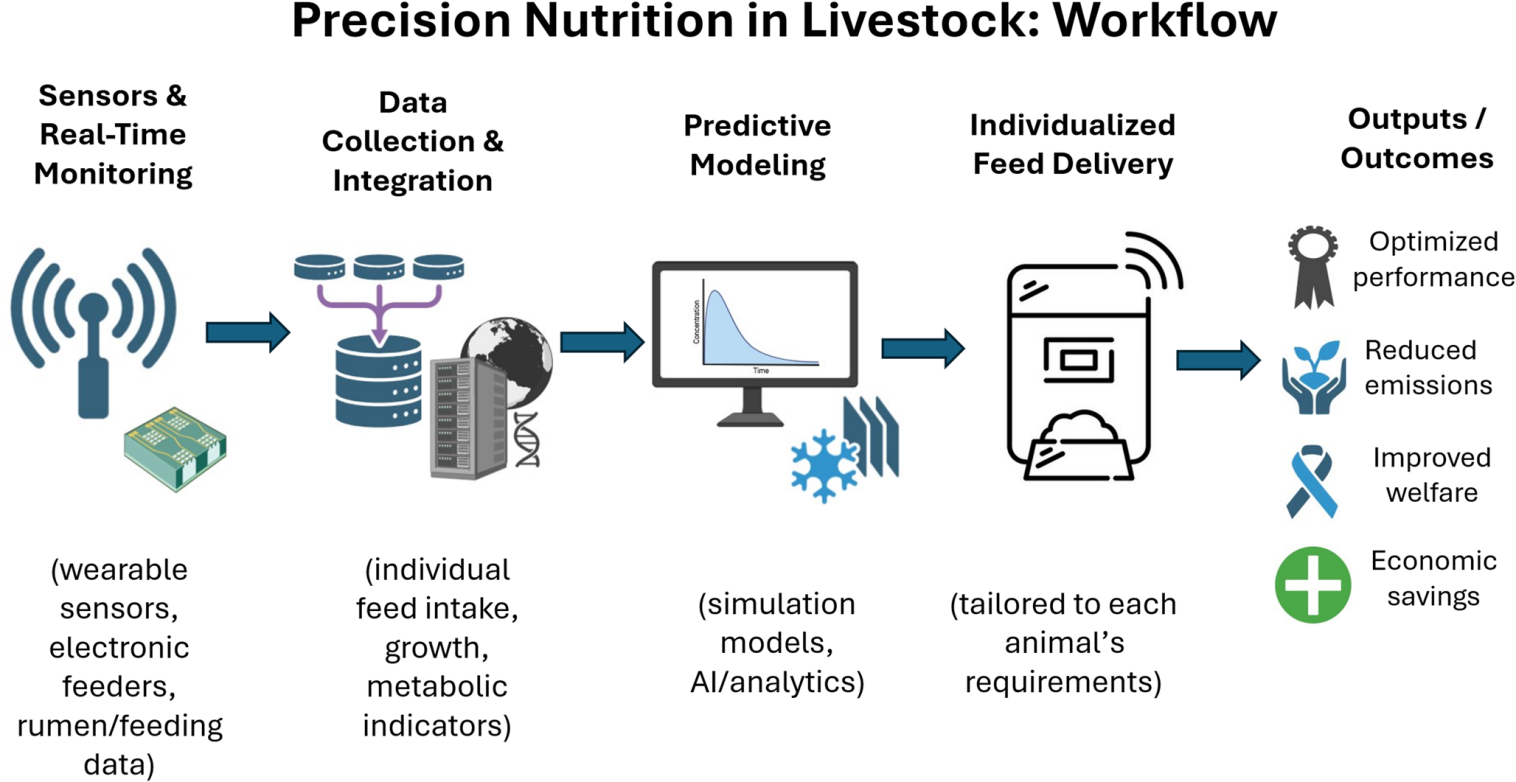
Conceptual workflow of precision nutrition in livestock systems. Real-time sensor data are collected and integrated to characterize individual animal intake, growth, and metabolic status. Predictive modeling and analytics are used to estimate dynamic nutrient requirements and guide individualized feed delivery. The workflow illustrates how data-driven precision feeding supports improved performance, reduced environmental emissions, enhanced welfare, and economic efficiency

Figure 2 illustrates a unified, nutrition-centered workflow for precision feeding in livestock systems, highlighting how real-time sensor data are translated into actionable feeding decisions. The framework integrates animal-level phenotypic and metabolic data with predictive modeling to estimate dynamic nutrient requirements. By linking data collection, analytics, and individualized feed delivery, the figure demonstrates how precision nutrition moves beyond monitoring toward closed-loop decision support. This conceptual workflow clarifies the functional role of Big Data and AI as enabling tools rather than endpoints. Overall, the figure contributes to conceptual coherence by explicitly connecting data-driven technologies with core animal nutrition outcomes, including feed efficiency, environmental sustainability, welfare, and economic performance.

These practical gains in precision feeding are rooted in a long-standing evolution of animal nutrition science toward mechanistic and individualized modeling frameworks. Over more than a century, animal nutrition science has evolved from empirical feeding standards toward mechanistic models that explicitly describe nutrient utilization at the animal level, culminating in metabolizable protein (MP) systems developed in the late twentieth century (e.g., NRC, ARC, INRA, CSIRO). While these frameworks converge in predicting MP requirements for lactation, they diverge markedly for growth due to contrasting assumptions regarding feed intake regulation, microbial protein synthesis, growth patterns, and tissue composition, underscoring the biological heterogeneity that precision nutrition aims to capture (Tedeschi et al. 2015). Advances in real-time sensing, nutrition simulation, and data analytics now enable more accurate and individualized predictions of intake, nutrient utilization, and emissions, although existing nutritional models require further adaptation to fully exploit sensor-derived data (González et al. 2018). Importantly, future progress depends on hybrid approaches that integrate mechanistic knowledge with AI rather than relying solely on data-driven methods or incremental model updates (Tedeschi 2019). Supporting this paradigm, machine-learning techniques such as Gaussian process regression outperform conventional statistical models in predicting nutrient digestibility under limited data conditions (Fu et al. 2019), while individual-level modeling reveals substantial variability in nutrient requirements across animals and production stages, reinforcing the limitations of population-average feeding strategies (Gauthier et al. 2019).

Precision nutrition in pigs and dairy cattle has been widely associated with reductions in feed costs, dietary protein and phosphorus intake, nutrient excretion, and, in some cases, GHGs without compromising productivity (Chase and Fortina 2023; Gaillard et al. 2020; Morey et al. 2023; Pomar and Remus 2019). However, the magnitude and consistency of these benefits are strongly context dependent. Positive outcomes are most frequently reported under controlled experimental conditions or in technologically advanced farms with accurate individual intake monitoring, whereas results in heterogeneous commercial systems are more variable due to genetic diversity, health status, social interactions, sensor accuracy, and deviations from assumed growth or production trajectories. In pigs, such variability can diminish both economic and environmental gains and may lead to inconsistent welfare outcomes when altered feeding patterns disrupt normal behavior. In dairy systems, although improvements in nitrogen-use efficiency are generally robust, trade-offs with CH₄ and CO₂ emissions have been observed under certain feeding strategies, and many reported long-term benefits rely on simulation models or region-specific data, limiting generalizability to pasture-based or low-input systems (González et al. 2018; Morey et al. 2023). Collectively, these findings indicate that precision nutrition should be viewed not as a universally transferable solution, but as a system-specific, adaptive management approach whose effectiveness depends on continuous monitoring and explicit consideration of biological, environmental, and management variability.

## Sources of big data in animal nutrition

In the context of precision animal nutrition, Big Data sources are most valuable when they contribute directly to nutrient requirement estimation, feed formulation, and feeding decision-support systems. Accordingly, the data streams described in this section are considered primarily in terms of their relevance to precision feeding, where heterogeneous datasets are integrated to inform individualized nutrient delivery rather than serving as independent descriptive outputs (González et al. 2018; Pomar and Remus 2019; Tedeschi 2022).

### Genomic and breed-specific data

Breed-specific genomic and multi-omics data provide a powerful foundation for precision nutrition in livestock. Crossbred genomic models, which account for dominance, imprinting, and breed-specific effects, achieve higher prediction accuracy when combined with high-density SNP panels and realistic covariance structures (Stock et al. 2020). Studies integrating SNP detection methods (Delta, FST, In) with machine learning classifiers (KNN, SVM, RF, NB, ANN) demonstrate that optimal breed assignment, and thereby accurate estimation of breed-specific nutrient requirements, depends on both the number of informative SNPs and the detection-classification method pair, with high-density SNP panels consistently improving performance across multiple cattle breeds (Zhao et al. 2023). Multi-breed evaluation using random selective genotyping further enhances prediction of sex-limited and low-heritability traits, with gains of 24.4% for moderate heritability (h² = 0.3) and 17–20% for low heritability (h² = 0.1), while minimizing bias (Gowane et al. 2025). Integration of genomic selection with AI-driven models and advanced omics, including transcriptomics, proteomics, metabolomics, and epigenetics, enables prediction of nutrition-relevant traits such as feed efficiency, milk composition, and metabolic resilience, supporting data-driven, climate-precision nutrition strategies and accelerated genetic improvement (Ahmad et al. 2023; Džermeikaitė et al. 2025). Systems that combine seedstock and commercial animal data further enhance selection accuracy for traits of nutritional and economic importance (Spangler et al. 2025). These genomic and multi-omics approaches, along with their key findings, are summarized in Table 1.

**Table 1:** Overview of genomic, phenotypic, and sensor-based data sources for precision nutrition, growth, and health monitoring in livestock. The table summarizes the technologies, traits measured, applications in nutritional management, key findings, and associated references based strictly on published studies

Data Source	Technology/Tool	Parameters Measured	Application in Precision Nutrition	Key Findings / Advantages	Reference
Genomic & Multi-omics	Crossbred genomic models, SNP panels	Feed efficiency, milk composition	Predict breed-specific nutrient requirements	Higher prediction accuracy with high-density SNP & realistic covariance structures	Stock et al. 2020
SNP Detection & ML	Delta, FST, In + KNN, SVM, RF, NB, ANN	Breed assignment	Supports accurate estimation of breed-specific nutrient requirements	Accuracy depends on SNP number and detection-classification method; high-density SNP panels improve performance	Zhao et al. 2023
Multi-breed Genotyping	Random selective genotyping	Sex-limited and low-heritability traits	Enhances prediction of traits relevant to nutrition and production	Gains of 24.4% for moderate heritability (h² = 0.3) and 17–20% for low heritability (h² = 0.1); minimizes bias	Gowane et al. 2025
Multi-omics Integration	Genomics + Transcriptomics, Proteomics, Metabolomics, Epigenetics	Feed efficiency, milk composition, metabolic resilience	Enables data-driven, climate-smart feeding strategies and genetic improvement	Supports prediction of nutrition-relevant traits and functional genome annotation	Ahmad et al. 2023; Džermeikaitė et al. 2025
Nutritional Databases	Dairy Cattle Nutrition & Feed Calculator	Dry matter intake, crude protein, total digestible nutrients	Formulates least-cost, nutrient-balanced rations	Android-based, integrates regression and linear programming	Patil et al. 2021
Large-Scale Feed Records	Feed composition database	25–30 nutrients across 174 feed types	Links nutritional intake with feed composition	Standardized 1.48 million records for accurate, large-scale feed composition	Tran et al. 2020
Feed Evaluation Systems	INRA & NRC	Duodenal nonammonia nitrogen, milk yield, OM/NDF digestibility, milk protein and lactose	Supports precise diet formulation and production efficiency	INRA offers better predictions for OM and NDF digestibility; higher concordance for milk lactose and protein than fat yield	Daniel et al. 2020
Smart Phenotyping	Wearable sensors, robotic milking, image-based phenotyping	Feeding behavior, activity, rumen conditions, body condition	Real-time monitoring of growth, feed efficiency, welfare, and disease resilience	High-throughput, cost-efficient characterization of complex traits	Dayoub et al. 2024; Neethirajan 2020; Neethirajan and Kemp 2021b; Siberski-Cooper and Koltes 2021
IoT & Wearables	Biometric wireless sensors (ear tags, collars, rumen boluses)	Activity, feeding, rumen conditions	Continuous monitoring for nutrition and welfare management	Enables remote monitoring and proactive management	Lee and Seo 2021; Neethirajan 2017
Advanced IoT	GPS-enabled collars, drones, nano-based diagnostics	Location, early disease biomarkers, welfare	Analytics-driven management of nutrition and health	Supports early disease detection, improves productivity; low-power, long-duration monitoring	Casas et al. 2021; Gehlot et al. 2022; Karthick et al. 2020

Here, SNP: single nucleotide polymorphism; ML: machine learning; KNN: k-nearest neighbor; SVM: support vector machine; RF: random forest; NB: naïve Bayes; ANN: artificial neural network; OM: organic matter; NDF: neutral detergent fiber; INRA: Institut National de Recherche pour l’Agriculture, l’Alimentation et l’Environnement; NRC: National Research Council; IoT: Internet of Things; GPS: Global Positioning System

From a nutritional perspective, genomic and multi-omics datasets are most informative when linked to traits directly influencing feed efficiency, nutrient partitioning, metabolic resilience, and responsiveness to dietary interventions. Their integration into precision feeding frameworks enables the differentiation of nutrient requirements among breeds, genotypes, and production stages, thereby supporting data-driven adjustment of diet composition rather than generalized ration formulation (Ahmad et al. 2023; Džermeikaitė et al. 2025; González et al. 2018).

### Nutritional intake and feed composition databases

Global intake of animal-source foods in 2018 showed substantial variation, with unprocessed red meat at 51 g/day, processed meat 17 g/day, seafood 28 g/day, eggs 21 g/day, milk 88 g/day, cheese 8 g/day, and yoghurt 20 g/day, with higher consumption observed among adults, urban residents, and populations with higher educational attainment (Miller et al. 2022). The growing complexity of livestock production and nutrition has driven the adoption of Big Data tools to capture, standardize, and analyze these diverse datasets. For instance, the Android-based Dairy Cattle Nutrition and Feed Calculator integrates regression-based nutrient prediction with linear programming to formulate least-cost rations by estimating dry matter intake, crude protein, and total digestible nutrients (Patil et al. 2021). Similarly, Tran et al. (2020) analyzed 2.76 million feed records, standardizing 174 feed types across 25–30 nutrients and refining 1.48 million records to construct accurate, large-scale feed composition databases. These examples highlight the critical role of big datasets in linking animal nutritional intake with feed composition, thereby supporting sustainable livestock management.

High-quality feed ingredient databases are fundamental for precise diet formulation and production efficiency. Achieving this requires standardized sampling procedures, representative collection methods (e.g., slotted grain probes), systematic sampling across multiple lots, and rigorous statistical determination of sample size, for example, 15 samples are sufficient to estimate soybean meal crude protein within ± 0.5% at 95% confidence (SD = 0.99) (Gonçalves et al. 2016). Modern feed evaluation systems, such as INRA (2018) and NRC (2001), provide reliable predictions of duodenal nonammonia nitrogen flow and milk yield. While both systems demonstrate comparable accuracy, INRA offers superior predictions for organic matter and neutral detergent fiber digestibility and higher concordance for milk lactose and protein than fat yield (Daniel et al. 2020).

Despite decades of development in feed composition tables, challenges persist. Managing large, heterogeneous datasets requires careful handling of misclassifications, accurate assessment of fiber fractions (total dietary fiber versus conventional methods), and integration of novel feed sources such as insects, algae, and single-cell proteins, which often exhibit high compositional variability (Schlageter-Tello et al. 2020). Digital tools, such as the Web-Based Ration Balancing Tool (WBRB), a free HTML–PHP–MySQL application, improve diet optimization efficiency and minimize errors compared with manual or spreadsheet-based methods (Radivojević et al. 2025). Moreover, the lack of harmonized feed consumption databases in regions such as Europe emphasizes the need for integrated models that link animal performance, feed composition, and consumption data. These models should be validated through case studies and aligned with established frameworks, including the European Catalogue of Feed Materials and FoodEx2, to enable accurate and reliable dietary assessments under real-world conditions (Pinotti et al. 2024). Key examples of databases, tools, and their applications are detailed in Table 1.

When incorporated into precision feeding systems, large-scale feed composition and intake databases support dynamic ration formulation, continuous updating of nutrient supply, and improved matching of dietary inputs with animal requirements. These databases form a critical bridge between analytical feed characterization and real-time feeding decisions, particularly when combined with predictive models that account for intake variability, production level, and physiological status (Daniel et al. 2020; Tedeschi 2022; Tran et al. 2020).

### Phenotypic data: growth, production, and health

The integration of smart livestock technologies, including wearable sensors, remote monitoring, robotic milking, and image-based or rumen phenotyping, enables high-throughput, real-time collection of behavioral, physiological, and production-related data, offering precise, species-specific insights into growth, feed efficiency, welfare, and disease resilience (Dayoub et al. 2024; Neethirajan 2020; Neethirajan and Kemp 2021b; Siberski-Cooper and Koltes 2021). These digital phenotyping approaches, combined with genomic selection and pan-genomic resources, facilitate cost-efficient characterization of complex traits, functional genome annotation, and development of heritable, biologically meaningful welfare and productivity traits, including stress adaptation, social behavior, and disease resistance (Brito et al. 2020; Klingström et al. 2025). While offering transformative potential for precision breeding and sustainable livestock production, their widespread adoption depends on standardized platforms, repeatable measurements across farms, durability under environmental stressors, and effective farmer education to ensure long-term implementation and data-driven management (Dayoub et al. 2024; Neethirajan and Kemp 2021b). A summary of the technologies, traits measured, and their applications is presented in Table 1.

In precision feeding applications, phenotypic data streams are most valuable when translated into nutritional indicators, such as changes in feed intake patterns, growth efficiency, metabolic status, or production responses to diet. The integration of these data into feeding models enables timely dietary adjustments, supporting individualized nutrient delivery while maintaining performance and welfare (Brito et al. 2020; González et al. 2018; Siberski-Cooper and Koltes 2021).

### Wearables, sensors, and IoT technologies

Wearable sensors and Internet of Things (IoT) technologies are transforming PLF by enabling real-time monitoring, health management, and data-driven decision-making in livestock production. Biometric and wearable wireless sensor systems (WWSS), including ear tags, collars, halters, rumen boluses, and tail- or vaginal-mounted devices, allow continuous tracking of physiological and behavioral parameters such as feeding, activity, and rumen conditions, although accuracy varies across measures, highlighting the need for standardized evaluation (Lee and Seo 2021; Neethirajan 2017). IoT integration of biological and environmental data via cloud platforms supports remote monitoring, reduces labor demands, and facilitates proactive management, yet requires species-specific sensor designs and faces challenges in data integration, large-scale validation, and system efficiency (Iwasaki et al. 2019; Neethirajan and Kemp 2021a). Emerging nano-based diagnostics, inertial sensors, GPS-enabled devices, drones, and edge computing further enhance early disease detection, animal welfare, and productivity, offering low-power, long-duration monitoring across rugged terrains (Casas et al. 2021; Gehlot et al. 2022; Karthick et al. 2020).Collectively, these technologies enable transformation from reactive to analytics-driven decision-making, improving feed efficiency, milk quality, traceability, and overall sustainability of livestock systems, although widespread adoption remains constrained by technological maturity and integration challenges (Akhigbe et al. 2021). Key devices, parameters measured, and practical applications are summarized in Table 1.

Within nutrition-centered precision livestock systems, sensor- and IoT-derived data acquire functional value when they inform feed intake estimation, nutrient utilization efficiency, and real-time feeding adjustments. Rather than serving solely as monitoring tools, these technologies enable the continuous feedback required for adaptive feeding strategies, linking animal responses to diet formulation and nutrient delivery in automated or semi-automated feeding systems (Neethirajan and Kemp 2021b; Pomar and Remus 2019; Tedeschi 2022).

## Machine learning approaches for precision nutrient management

In animal nutrition, machine learning approaches are most valuable when they directly support precision feeding systems by improving the estimation of nutrient requirements, predicting feed efficiency, and optimizing diet formulation. Accordingly, the applications reviewed in this section are evaluated based on their contribution to feeding decision-support frameworks, in which predictive models translate established nutritional principles into real-time, individualized feeding strategies rather than replace them (González et al. 2018; Pomar and Remus 2019; Tedeschi 2022).

### Machine learning for animal-level nutrient prediction

Between 2020 and 2025, diverse machine learning (ML) algorithms, including linear and logistic regression, tree-based methods, support vector machines, neural networks, and clustering approaches, have been increasingly applied to model nutrient utilization and predict performance in livestock systems (Grzesiak et al. 2025). Neural networks and ensemble methods such as random forest and gradient boosting have outperformed traditional regression approaches in predicting nitrogen excretion, milk yield, and daily nutrient requirements in dairy cows and gestating sows, demonstrating higher accuracy, lower error, and strong correlations with measured outcomes (Chen et al. 2022; Durand et al. 2023; Nguyen et al. 2020; J. Su et al. 2025a, b). Autoregressive models and support vector machines have shown superior forecasting of lactational performance under variable nutritional conditions, while Bayesian networks, spline regression, and Gaussian process models have revealed complex non-linear interactions between diet composition, production traits, and CH₄ emissions, highlighting critical relationships for sustainable nutrient management (Mohammadabadi et al. 2025). These advancements, supported by hybrid mechanistic and data-driven modeling, real-time sensor data, and open-source programming tools, have facilitated enhanced predictive accuracy and knowledge-based precision feeding at the animal level (Brennan et al. 2023; Ennaji et al. 2023).

### Machine learning for landscape-scale nutrient and forage management

ML applications have also expanded to landscape- and farm-level nutrient management, integrating remote sensing, UAV imagery, hyperspectral analysis, and socio-environmental datasets to predict pasture biomass, forage quality, and nutrient uptake with high precision (De Rosa et al. 2021; Fernandes & Tedeschi, 2025; Saha et al. 2025; Serpa-Imbett et al. 2025). Random forest, generalized additive models, and deep learning approaches have achieved R² values up to 0.998 for biomass and quality estimation, while identifying key environmental and management variables influencing nutrient availability, grazing frequency, and soil fertility (Nikoloski et al. 2019; Wang et al. 2025a, b). The biological relevance of such landscape-scale nutritional heterogeneity is underscored by evidence from free-ranging herbivores, where spatial variation in digestible forage biomass-driven primarily by fire-mediated shifts in plant community composition rather than forage digestibility or phenology, was associated with reduced autumn body fat, lower pregnancy rates (72% vs. 89%), and increased demographic vulnerability in adult female elk occupying nutritionally constrained summer ranges compared with those in higher-quality habitats (Proffitt et al. 2016). Together, these findings demonstrate how spatially explicit nutritional constraints can translate directly into population-level performance outcomes. Integrated ML frameworks further enable the identification of unregistered animal feeding operations and the assessment of nutrient-related environmental risks, supporting evidence-based nutrient management across production systems (Saha et al. 2025). Despite these advances, challenges remain in developing hybrid diagnostic–prescriptive models, integrating behavioral, health, and soil variables, and translating ML predictions into actionable feed formulation strategies, emphasizing the need for transparency, reproducibility, and mechanistic understanding in PLF (García et al. 2020; Tedeschi 2022).

**Fig. 3:**
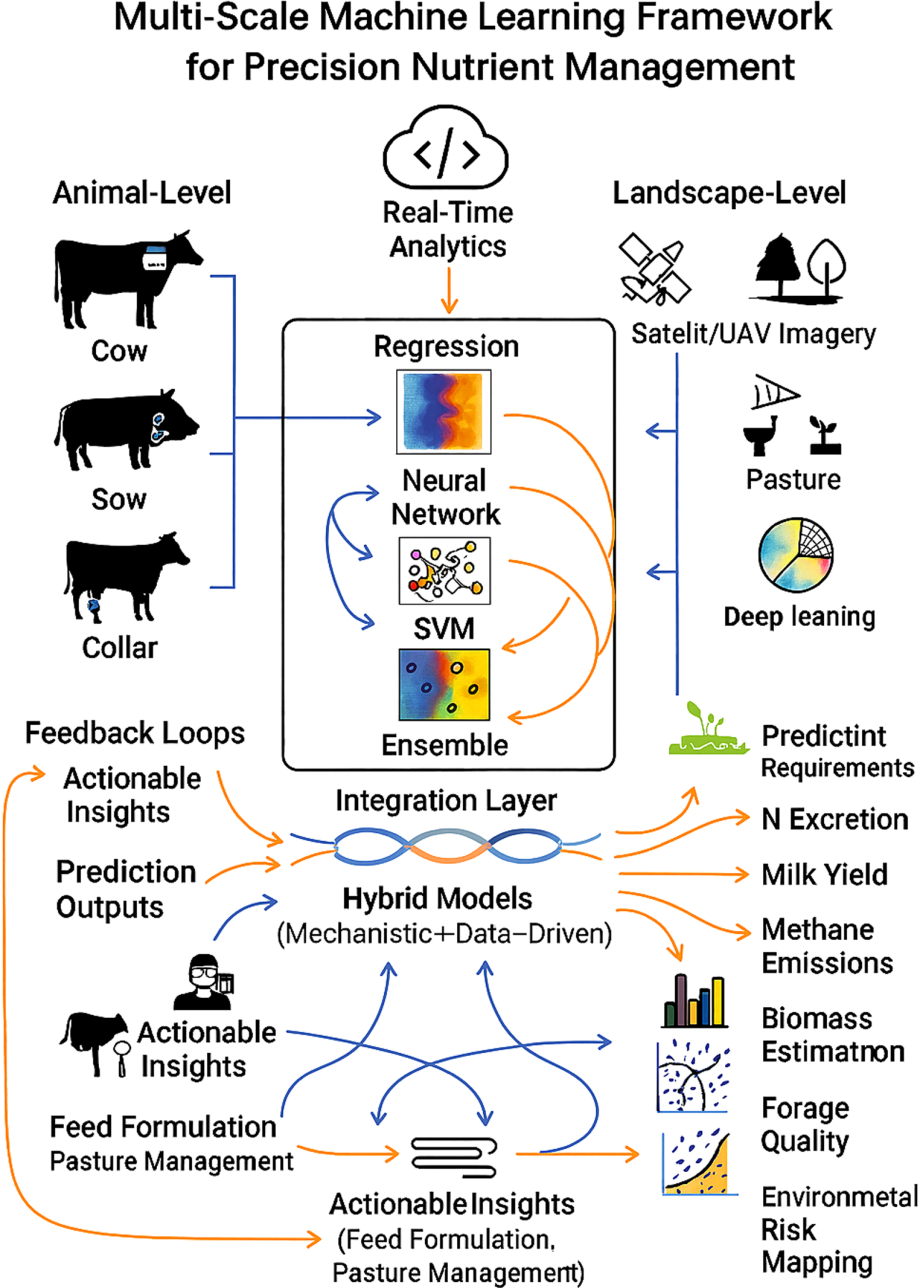
Conceptual schematic of a multi-scale machine learning framework that integrates animal-level sensor data with landscape-level environmental inputs to enhance precision nutrient management in livestock systems. Predictive algorithms estimate outputs including nutrient requirements, nitrogen emissions, and forage quality. A hybrid integration layer supports real-time analytics and feedback mechanisms, enabling adaptive decision-making for feed formulation and pasture optimization

Building on these landscape-level advances, ML models have also shown superior predictive performance over traditional regression approaches for animal-level nutrient requirements, feed efficiency, and production traits (Chen et al. 2022; Durand et al. 2023; Shirzadifar et al. 2025). However, the transition from predictive accuracy to practical robustness remains challenging. Most models rely on historical datasets and are validated under relatively static conditions, with limited evidence of reliable performance under real-time farm variability, including health disturbances, dietary transitions, or climatic stressors (Palma et al. 2025). Moreover, increasing algorithmic complexity often reduces interpretability, constraining transparency, user trust, and adoption, while models developed for specific breeds or management systems may lack transferability and risk overfitting (García et al. 2020; Tedeschi 2022). These limitations underscore the need for coherent, multi-scale frameworks, such as the integrated approach illustrated in Fig. 3, that presents a multi-scale ML framework that integrates animal-level and landscape-level data to support precision nutrient management in livestock systems. By combining real-time analytics with diverse modeling approaches, the framework illustrates how heterogeneous data streams are translated into nutrition-relevant predictions. The inclusion of hybrid mechanistic and data-driven models highlights a pathway for improving biological interpretability alongside predictive accuracy. Importantly, the figure demonstrates how model outputs are converted into actionable feeding and pasture management decisions. This integrative perspective advances understanding of how ML can be operationalized to improve nutrient efficiency, environmental sustainability, and production performance.

## Integration of omics technologies and AI

### Nutrigenomics and precision diet design

**Fig. 4:**
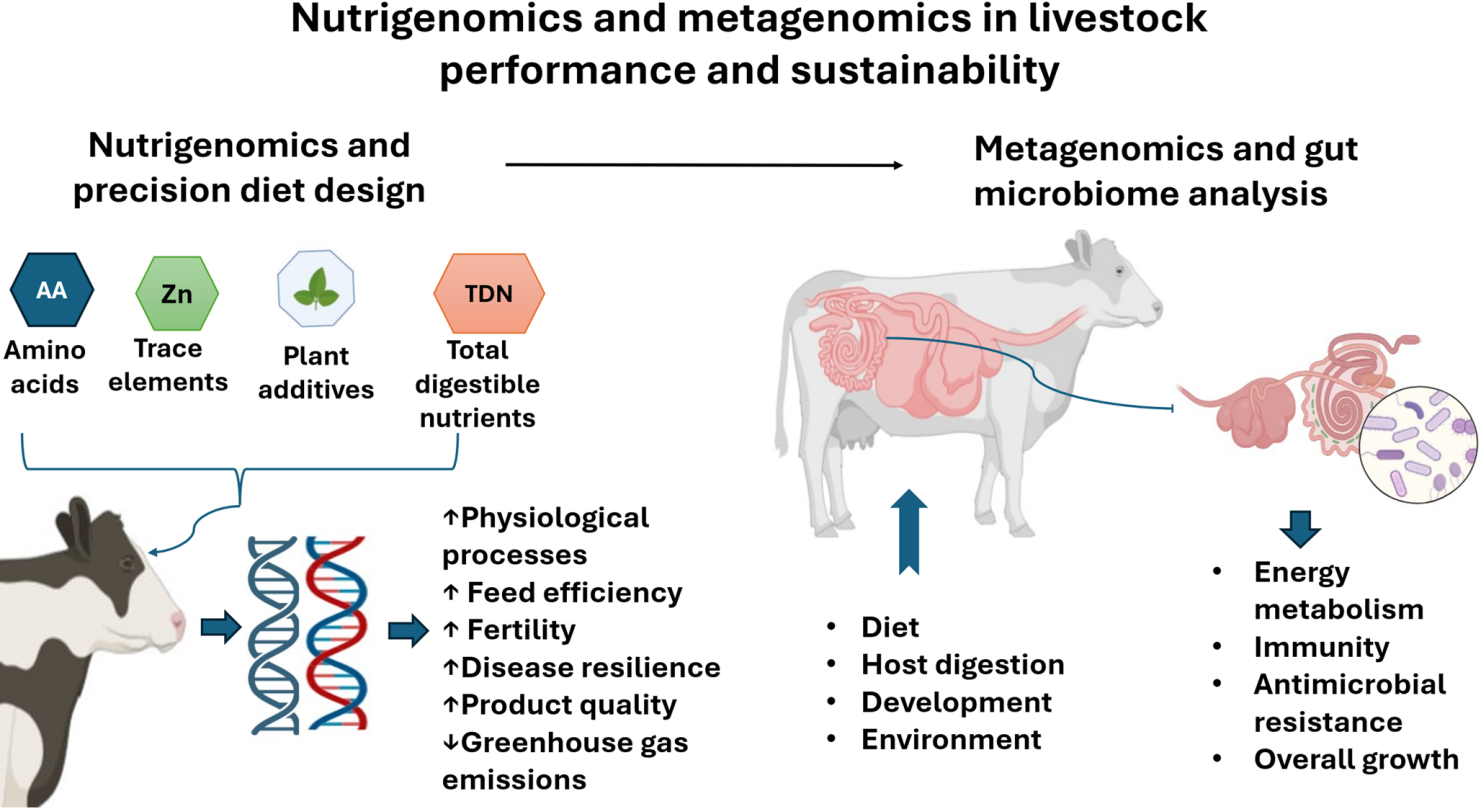
Conceptual integration of nutrigenomics and metagenomics in precision livestock nutrition. Dietary components modulate host gene expression and physiological processes influencing feed efficiency, fertility, and product quality. Concurrently, gut microbiome composition affects energy metabolism, immunity, antimicrobial resistance, and growth. The framework highlights host–diet–microbiome interactions as a basis for sustainable livestock performance

Ruminant nutrigenomics enables the integration of genetic profiles with tailored diets to enhance productivity, feed efficiency, product quality, disease resilience, and fertility, while reducing GHGs emissions (Kizilaslan et al. 2022). Livestock cells sense nutrients, including fatty acids, amino acids, trace elements, and overall energy intake, modulating gene expression, metabolic pathways, and tissue function (Loor 2022). High throughput ‘omics’ approaches have elucidated intricate regulatory networks governing these physiological responses, offering a foundation for precision nutrition. Although application in livestock, particularly pigs, is limited by cost, long generation intervals, and ethical considerations, evidence indicates that dietary adjustments, such as variations in fat, protein, microelements, and plant-derived additives, affect gene expression, protein synthesis, and epigenetic mechanisms across prenatal, neonatal, and adult stages (Nowacka-Woszuk 2020). In ruminants, genotype-informed strategies show measurable benefits: Hanwoo steers grouped by marbling score estimated breeding value (MS-EBV) achieved ~ 20% more top-quality carcasses, while dietary total digestible nutrient levels had minimal impact and no genotype-by-nutrition interaction (Gajaweera et al. 2023). Similarly, transitioning Jersey steers between high-forage and high-concentrate diets influenced body weight and modulated expression of genes linked to fatty acid metabolism (ACADSB) and growth (AKT3, IGFBP5), without altering ruminal papillae morphology (Novak et al. 2019). These findings highlight the potential of integrating genomics with diet design to optimize performance and sustainability in livestock systems. Figure 4 conceptualizes the complementary roles of nutrigenomics and metagenomics in advancing precision nutrition for livestock performance and sustainability. The framework illustrates how diet composition influences host gene expression, physiological processes, and production traits, while parallel modulation of the gut microbiome governs energy metabolism, immunity, and antimicrobial resistance dynamics. By integrating host–diet–microbiome interactions, the figure highlights a systems-level approach to improving feed efficiency and product quality. Importantly, it demonstrates how molecular-level insights can be translated into practical diet design strategies. This integrative perspective contributes to a deeper understanding of nutrition-driven pathways for sustainable livestock production.

From a precision feeding perspective, nutrigenomic information is most valuable when translated into dietary adjustments that improve nutrient efficiency and production outcomes, rather than serving as a standalone genomic characterization (Kizilaslan et al. 2022; Loor 2022).

### Metagenomics and the gut microbiome

**Fig. 5:**
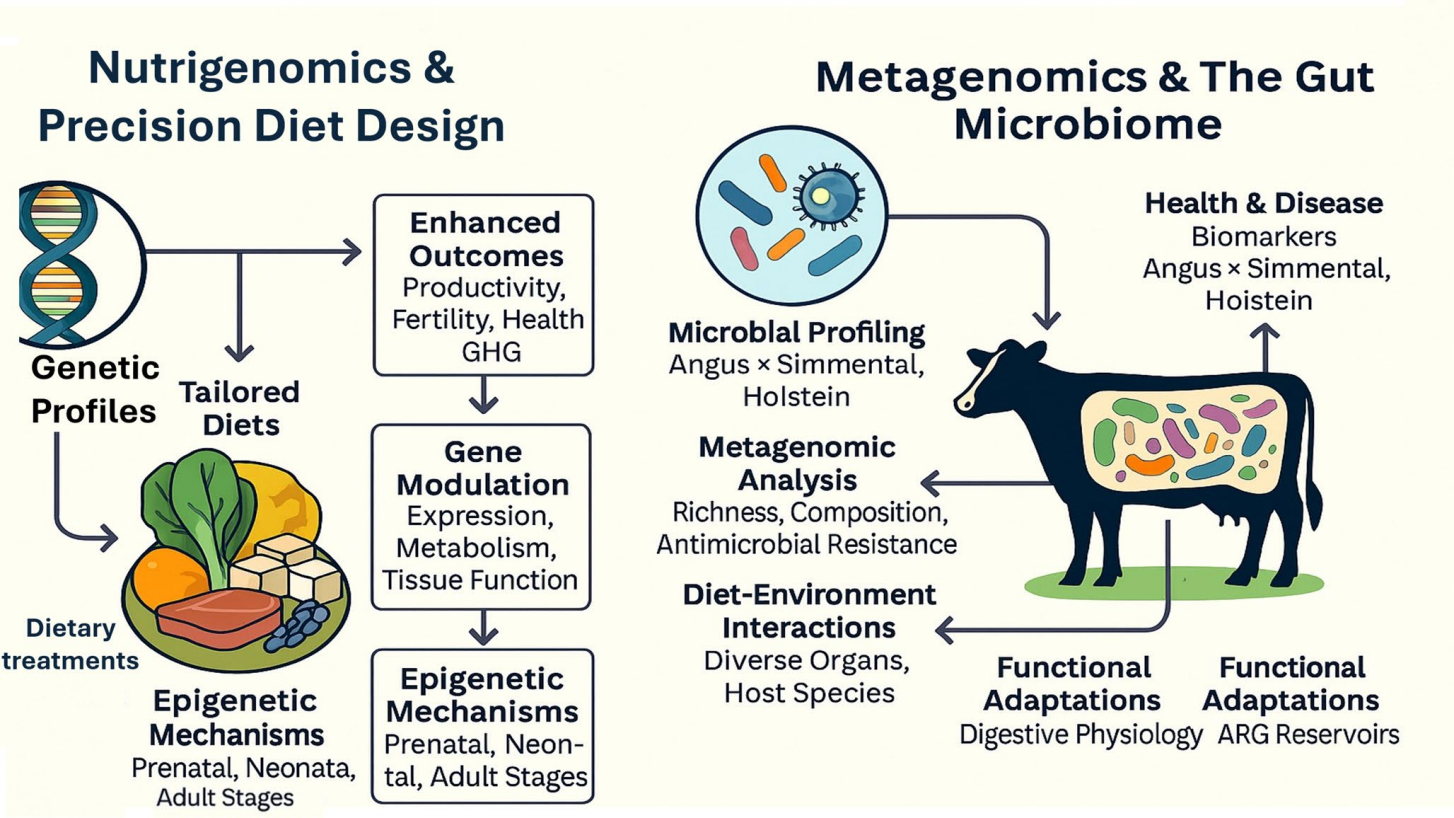
Integrated framework of nutrigenomics and metagenomics in precision livestock nutrition. Genetic profiles and targeted dietary interventions regulate gene expression, metabolism, and epigenetic mechanisms across developmental stages. Concurrently, gut microbiome composition and function mediate digestive physiology, immunity, and antimicrobial resistance. The framework illustrates how host–diet–microbiome interactions can be leveraged to improve productivity, health, and sustainability in livestock systems

Metagenomics has revolutionized livestock research by enabling comprehensive profiling of microbial communities, their functions, and antimicrobial resistance genes (ARGs) across diverse organs and host species (Kumar 2025). Firmicutes taxa, including *Acetivibrionaceae*,* Clostridiaceae*,* Lachnospiraceae*,* Ruminococcaceae*, and CAG-74, are evolutionarily conserved across vertebrate gut metagenomes, spanning ruminants, monogastrics, and humans, with prevalence linked to pathways supporting host colonization, survival, and transmission (Dias et al. 2025). Host digestive physiology shapes microbiota composition and function, with specialized adaptations observed in cattle, sheep, goats, pigs, horses, rabbits, and chickens (Tardiolo et al. 2025). Metagenomic analyses identify microbial signatures associated with health and disease: fescue toxicosis in Angus × Simmental cattle reduces diversity and enriches *Ruminococcaceae* bacterium P7, a potential herd-level biomarker (Alfaro et al. 2025), while endometritis in Holsteins elevates Fusobacterium and alters Wnt/catenin pathways (Rashid et al. 2025). Diet and environment further modulate microbiota, influencing energy metabolism, immunity, and growth, as shown in pigs (Tang et al. 2025), plateau pikas (J.-W. Su et al. 2025a, b), and rats fed Tenebrio molitor meal (Gałęcki et al. 2025). Gut microbiomes also act as ARG reservoirs, with frequent horizontal gene transfer across human, avian, and environmental niches, highlighting their role in antimicrobial resistance dissemination (Napit et al. 2025). Figure 5. illustrates an integrated framework linking nutrigenomics-based precision diet design with metagenomic analysis of the gut microbiome in livestock. The figure demonstrates how genetic profiles and dietary interventions modulate gene expression, metabolic pathways, and epigenetic mechanisms across developmental stages. In parallel, microbiome profiling reveals diet–environment interactions that influence digestive function, immunity, and antimicrobial resistance. By connecting host genomic responses with microbial functional adaptations, the framework highlights a systems-level approach to improving productivity, health, and sustainability. This integrative view advances understanding of how molecular nutrition can be translated into targeted feeding strategies in modern livestock systems.

Building on these insights, nutrigenomics and metagenomics provide a powerful conceptual framework for understanding host–diet–microbiome interactions and their implications for feed efficiency, product quality, and environmental sustainability (Dias et al. 2025; Loor 2022). However, translating this knowledge into actionable precision nutrition strategies remains challenging. Genotype-informed dietary optimization has shown the greatest promise under controlled experimental conditions, whereas robust genotype-by-nutrition responses in commercial systems are less consistently demonstrated (Gajaweera et al. 2023; Nowacka-Woszuk 2020). Similarly, although metagenomic studies reveal strong diet–microbiome associations, high inter-individual variability, environmental confounding, and system complexity constrain causal inference and predictive accuracy at the farm level (Tang et al. 2025; Tardiolo et al. 2025). High analytical costs, data management demands, and ethical considerations further limit widespread adoption. Consequently, omics-based approaches currently function most effectively as integrative tools for hypothesis generation and system-level understanding rather than as standalone prescriptive solutions, emphasizing the need for scalable, cost-effective validation across diverse livestock production systems.

While metagenomic analyses also provide important insights into antimicrobial resistance and microbial ecology, this review emphasizes these findings only insofar as they relate to diet-mediated modulation of the gut microbiome, nutrient utilization, and feed efficiency. Broader epidemiological or public health aspects of antimicrobial resistance are beyond the scope of this nutrition-focused review.

## Precision nutrition and precision delivery

Precision nutrition systems, powered by Big Data and AI, are driving a new era of precision animal nutrition by integrating automated feed delivery, real-time monitoring, and predictive decision-making. Leveraging IoT devices, ML algorithms, and advanced sensors, these systems optimize feed efficiency, reduce wastage, and enhance nutrient utilization, supporting both productivity and sustainability goals (Kala et al. 2025; Neculai-Valeanu et al. 2025). Automated feeders demonstrate precise feed placement, effective obstacle navigation, and energy-efficient operation, while simulation studies identify design parameters that maintain feed rate errors below 5% (Gao et al. 2025; Raut et al. 2025). Beyond mechanized delivery, smart sensors capture detailed information on animal behavior, health, and social interactions, enabling real-time adjustments that improve welfare, though integration with environmental control systems remains limited (Bordignon et al. 2025; Seger et al. 2025). AI-enhanced platforms, including walk-over-weighing systems and heat stress management tools, provide non-invasive, high-accuracy monitoring, detect anomalies proactively, and support measurable improvements in production, animal health, and environmental resilience at minimal cost (Islam and Khan 2025; Kirbas 2025; Vedhashree et al. 2025). Collectively, these innovations exemplify a paradigm shift in precision livestock feeding, where intelligent automation, predictive analytics, and behavioral insights converge to optimize nutrition, welfare, and sustainable production across diverse livestock systems. By bridging technology and animal-centered management, these systems establish a practical, scalable framework for the future of precision feeding.

Building on these AI-enabled precision nutrition, big data-driven precision feeding has emerged as a pivotal response to mounting pressures on global food systems arising from population growth, dietary shifts toward animal-source foods, land constraints, and climate change (Balakuntala et al. 2018). Under precision nutrition and precision delivery, such approaches leverage advanced sensors, automation, and data analytics to enable real-time, individualized nutrient supply, thereby improving feed efficiency while simultaneously reducing feed waste, nutrient excretion, GHGs emissions, and water pollution, with concurrent benefits for animal welfare (Sonea et al. 2023). When embedded within circular-economy frameworks, precision feeding can further amplify sustainability outcomes by closing nutrient loops across livestock and crop systems. Evidence from integrated closed-loop agricultural models demonstrates that recycling nutrients from organic waste streams through anaerobic digestion, biochar production, and nutrient recovery, can reduce reliance on mineral fertilizers, enhance resource efficiency, and strengthen farm resilience at relatively low cost (McConville et al. 2015; van der Velden et al. 2022). Complementary advances in nutrient adsorption and controlled-release technologies, such as high-capacity biochar-based urea recovery systems, further illustrate how data-enabled nutrient management can translate recycled resources into tangible productivity gains, reinforcing the role of precision nutrition as a cornerstone of sustainable, precision-oriented animal nutrition (Ganesapillai et al. 2015; Sonea et al. 2023).

Collectively, these systems enable the practical implementation of precision feeding by integrating real-time animal responses with automated nutrient delivery, thereby closing the loop between nutritional modeling and on-farm feeding management.

## Future directions and research gaps

Although AI-enabled precision feeding has significantly advanced livestock nutrition, several critical knowledge and implementation gaps remain. Future studies should prioritize fully integrating behavioral, physiological, and environmental monitoring to enable adaptive, real-time feeding systems. The development of advanced predictive models that incorporate genomic information, gut microbiome composition, and multi-source physiological indicators offers substantial potential for refining individualized nutrition strategies. However, translating these complex datasets into practical and interpretable decision-support tools remains a major challenge. Greater model transparency and rigorous biological validation are essential to ensure reliability under commercial production conditions. In addition, Long-term assessments of economic, environmental, and sustainability impacts are limited, and issues related to data interoperability, sensor standardization, and cybersecurity must be addressed. Interdisciplinary collaboration between animal scientists, engineers, and data analysts is critical to realize the full potential of precision feeding as a tool for sustainable, efficient, and resilient livestock production.

## Ethical, legal and governance challenges of AI in animal nutrition

The rapid integration of Big Data and AI into animal nutrition and precision feeding raises interconnected ethical, legal, and governance challenges that must be addressed to ensure responsible innovation. As the first binding, cross-sectoral regulatory framework with extraterritorial reach, the EU Artificial Intelligence Act establishes a global benchmark for trustworthy AI, with relevance for data-intensive domains such as precision animal nutrition, where concerns over animal welfare, producer rights, and algorithmic accountability are increasingly salient (González et al. 2018; Smuha 2025). PLF has emerged as a response to growing global demand for animal products, larger herd sizes, and declining labor availability, enabling continuous, real-time monitoring of animal health, welfare, productivity, and environmental impact beyond what traditional retrospective assessments can achieve (Berckmans 2017). While such continuous surveillance can enhance early disease detection and management efficiency, it also raises ethical concerns that productivity-driven optimization and pervasive monitoring may undermine natural behaviors and welfare unless AI-driven decisions are explicitly aligned with genuine welfare objectives (Schillings et al. 2021). Moreover, the digitalization of agriculture reshapes power relations within agri-food systems by intensifying debates around data ownership, privacy, and control, underscoring the need for interdisciplinary governance approaches that critically assess both the promises and structural limitations of data-driven farming (Bronson and Knezevic 2016). These concerns are compounded by evidence that AI systems deployed in high-stakes contexts frequently reproduce bias and unfair outcomes, with no universal fairness solution available, highlighting the necessity of domain-specific, systematically evaluated governance frameworks (Mehrabi et al. 2021). Within this context, PLF technologies offer a transformative opportunity to improve animal welfare holistically, beyond health and productivity alone, provided their development is guided by multi-actor collaboration and robust regulatory oversight, a role the EU AI Act begins to operationalize through enforceable transparency and accountability mechanisms despite remaining practical and institutional challenges (Buller et al. 2020; Söderlund 2025).

## Conclusion

The integration of Big Data, AI, and multi-omics technologies is fundamentally reshaping precision animal nutrition. These innovations enable individualized, evidence-based feeding strategies that enhance production efficiency while promoting animal welfare and environmental sustainability. The use of real-time monitoring systems, advanced predictive models, and adaptive nutrient delivery mechanisms demonstrates substantial potential across diverse livestock production systems. Addressing challenges in data integration, model standardization, and scalability through interdisciplinary collaboration will be critical to establishing AI-enabled precision feeding as a cornerstone of sustainable, resilient, and ethically responsible livestock production.

## Data Availability

Data is available on request.
